# Antimicrobial Activity of Essential Oils against *Staphylococcus* and *Malassezia* Strains Isolated from Canine Dermatitis

**DOI:** 10.3390/microorganisms8020252

**Published:** 2020-02-13

**Authors:** Valentina Virginia Ebani, Fabrizio Bertelloni, Basma Najar, Simona Nardoni, Luisa Pistelli, Francesca Mancianti

**Affiliations:** 1 Department of Veterinary Science, University of Pisa, Viale delle Piagge 2, 56124 Pisa, Italy; fabrizio.bertelloni@unipi.it (F.B.); simona.nardoni@unipi.it (S.N.); francesca.mancianti@unipi.it (F.M.); 2 Centro Interdipartimentale di Ricerca “Nutraceutica e Alimentazione per la Salute”, University of Pisa, via del Borghetto 80, 56124 Pisa, Italy; luisa.pistelli@unipi.it; 3 Department of Pharmacy, University of Pisa, via Bonanno 6, 56126 Pisa, Italy; basmanajar@hotmail.fr

**Keywords:** *Staphylococcus*, *Malassezia*, dogs, essential oils, antimicrobial activity

## Abstract

*Staphylococcus* spp. bacteria are the most frequently involved agents in canine cutaneous infections. Treatment of these infections is based on antibiotic therapy, that often is not effective because of the antibiotic-resistance of the bacterial strains. Cutaneous staphylococcal infections are often complicated by *Malassezia* yeasts, that may be resistant to the conventional antifungal drugs. The present investigation was aimed to evaluate the in vitro antimicrobial activity of some essential oils (EOs) in view of a potential cutaneous application. In detail, EOs obtained from lemon verbena (*Aloysia triphylla* L’Hèr. Britton), cinnamon (*Cinnamomum zeylanicum* J. Presl), myrrh (*Commiphora myrrha* (Nees) Engl. var. *molmol*), lemongrass (*Cymbopogon citratus* (DC.) Stapf), litsea (*Litsea cubeba* (Lour.) Pers.), lemon balm (*Melissa officinalis* L.), oregano (*Origanum vulgare* L.), savory (*Satureja* *montana* L.), and thyme (*Thymus vulgaris* L.) were assayed against *Staphylococcus* spp. and *Malassezia* *pachydermatis* strains previously isolated from dogs with dermatitis. All EOs were tested by agar disk diffusion and minimum inhibitory concentration methods to verify the antistaphylococcal activity, and by a microdilution method to evaluate the activity against *M. pachydermatis*. *O. vulgare, T. vulgaris*, and *S. montana* showed the best antibacterial activity against all the selected strains, with MICs ranging from 0.29 to 0.58 mg/mL, from 0.58 to 1.16 mg/mL, and from 0.56 to 1.12 mg/mL, respectively, whereas *A. triphylla* (1.03 mg/mL) and *S. montana* (1.8 mg/mL) were the most active against *M. pachydermatis*. After a proper in vivo evaluation, *O. vulgare*, *T. vulgaris*, and *S. montana* EOs could be a promising treatment to combat canine cutaneous mixed infections.

## 1. Introduction

Bacterial infections are responsible for the most frequent disorders of the skin of companion animals, mainly dogs. In particular, pyoderma is a pyogenic infection that can affect epidermis and hair follicle units or dermis and subjacent fatty tissue. Atopic dermatitis is a genetically predisposed inflammatory and pruritic allergic skin disease in which the skin microbiome may cause secondary infections that can influence its severity [1,2]. In both cases, Staphylococci are the most frequently involved agents: *S. pseudointermedius* is considered the primary canine cutaneous pathogen, but other *Staphylococcus* species may be found in skin infection, as well as *Escherichia coli*, *Proteus* spp., and *Pseudomonas* spp.

Treatment of canine bacterial skin infections is usually based on antibiotic therapy, which is often not effective because of the involvement of antibiotic-resistant bacterial strains.

*Malassezia* sp. are lipophilic yeasts, commensal of mammals’ skin, responsible for dermatitis/otitis in dogs. The overgrowth of these agents is recognized to trigger bacterial pyoderma [3]. Furthermore, *Malassezia* yeasts and *Staphylococcus* spp. are responsible for concurrent infection [4] in both canine and human atopic dermatitis, making the pharmacologic treatment a relevant tool in the patients’ management [5]. The diseases are well characterized by relapses, and in vitro studies report that yeasts cultured from clinical lesion are more resistant to antifungal drugs, when compared with organisms taken from asymptomatic subjects [6,7].

Essential oils (EOs) are volatile oils obtained from herbs, soluble in alcohol and ether but insoluble in water, with characteristic odors responsible for the scents that plants emit. They are widely employed in cosmetics industry, perfumery, and aromatherapy [8]. The antimicrobial properties of several EOs and their constituents have been studied mainly through assays against bacterial and fungal strains of different genera, including staphylococci [8,9,10,11,12].

The present investigation was aimed to evaluate the antimicrobial activity of some EOs, chosen for their not cytotoxic character, as reported by the producer, in view of a potential cutaneous application. In detail, EOs obtained from lemon verbena (*Aloysia triphylla* L’Hèr. Britton), cinnamon (*Cinnamomum zeylanicum* J. Presl), myrrh (*Commiphora myrrha* (Nees) Engl. var. *molmol*), lemongrass (*Cymbopogon citratus* (DC.) Stapf), litsea (*Litsea cubeba* (Lour.) Pers.), lemon balm (*Melissa officinalis* L.), oregano (*Origanum vulgare* L.), savory (*Satureja montana* L.), and thyme (*Thymus vulgaris* L.) were assayed against *Staphylococcus* spp. and *Malassezia pachydermatis* strains previously isolated from dogs with dermatitis.

## 2. Material and Methods

### 2.1. Essential Oils

Essential oils (EOs) from the following nine plants were employed in this study: lemon verbena (*Aloysia triphylla* L’Hèr. Britton), cinnamon (*Cinnamomum zeylanicum* J. Presl), myrrh (*Commiphora myrrha* (Nees) Engl. var. *molmol*), lemongrass (*Cymbopogon citratus* (DC.) Stapf), litsea (*Litsea cubeba* (Lour.) Pers.), lemon balm (*Melissa officinalis* L.), oregano (*Origanum vulgare* L.), savory (*Satureja montana* L.), thyme (*Thymus vulgaris* L.).

All EOs (FLORA^®^, Pisa, Italy), were maintained in dark glass vials at 4 °C until used in the different experiments.

Quality control for antibacterial and antimycotic activity was tested for each EO before the analyses. For this purpose, each EO was streaked onto a blood agar plate, and the plates were incubated at 37 °C for 48 hours. Absence of colonies after the incubation period confirmed the EOs sterility.

#### Essential Oils Analysis

The hydrodistilled essential oils were diluted to 0.5% in HPLC-grade n-hexane and then injected into a GC–MS apparatus. Gas chromatography–electron impact mass spectrometry (GC–EIMS) analyses were performed with an Agilent 7890B gas chromatograph (Agilent Technologies Inc., Santa Clara, CA, USA) equipped with an Agilent HP-5MS (Agilent Technologies Inc., USA) capillary column (30 m × 0.25 mm; coating thickness 0.25 μm) and an Agilent 5977B single quadrupole mass detector (Agilent Technologies Inc., USA). Analytical conditions were as follows: injector and transfer line temperatures of 220 and 240 °C, respectively; oven temperature programmed from 60 to 240 °C at 3 °C/min; carrier gas helium at 1 mL/min; injection of 1 μL (0.5% HPLC grade n-hexane solution); split ratio 1:25. The acquisition parameters were as follows: full scan; scan range: 30–300 *m*/*z*; scan time: 1.0 s. Identification of the constituents was based on a comparison of the retention times with those of the authentic samples, comparing their linear retention indices relative to the series of n-hydrocarbons. Computer matching was also used against commercial (NIST 14 and ADAMS) and laboratory-developed mass spectra library built up from pure substances and components of known oils and MS literature data [13,14,15,16,17,18].

### 2.2. Antibacterial Activity

#### 2.2.1. Bacterial Strains

A total of eight *Staphylococcus* spp. strains were tested in vitro for antimicrobial sensitivity. All strains were previously isolated from skin of dogs with dermatitis and typed using the API Staph system (BioMerieux, Milan, Italy). In detail, the isolates were 1 *S. aureus*, 1 *S. pseudointermedius*, 1 *S. hyicus*, 2 *S. chromogenes*, and 3 *S. xylosus*.

The isolates were kept in collection at −80 °C in glycerol broth. Each strain was inoculated into brain hearth infusion broth (BHIB, Oxoid Ltd., Basingstoke, Hampshire, UK) and incubated at 37 °C for 24 h. Cultures of 1–2 × 10^7^ CFU/mL, corresponding to 0.5 McFarland standard, were employed in the tests.

#### 2.2.2. Agar Disk Diffusion Method

Antibacterial activity of the selected EOs was tested by Kirby–Bauer agar disk diffusion method following the procedures reported by Clinical and Laboratory Standards Institute (CLSI) [19]. Briefly, EOs were 5% diluted in dimethyl sulfoxide (DMSO, Oxoid Ltd.), and one absorbent paper disk was impregnated with 10 µL of each dilution, respectively, and tested against each isolate.

A paper disk impregnated with 10 µL of DMSO was included as negative control. A commercial disk impregnated with chloramphenicol (30 µg) (Oxoid) was used as positive control. Growth inhibition zones were evaluated after incubation at 37 °C for 24 h. All tests were performed in triplicate.

The in vitro sensitivity of all *Staphylococcus* isolates to chloramphenicol (30 µg) (Oxoid) was assayed by the same method, and the results were interpreted as indicated by CLSI [20].

#### 2.2.3. Minimum Inhibitory Concentration

Minimum inhibitory concentration (MIC) was determined for all EOs with the broth microdilution method, following the guidelines of CLSI [21] and the protocol previously described [22]. Briefly, the test was performed in 96-well microtiter plates in a total volume of 200 µL/well including 160 µL of BHIB (Oxoid), 20 µL of each bacterial suspension, and 20 µL of each EO. The MIC value was determined as the lowest concentration, expressed in percentage and mg/mL, of each EO at which staphylococci show no visible growth. The same assay was performed simultaneously for bacterial growth control (tested agents and media) and sterility control (tested oil and media). Positive control using chloramphenicol (Oxoid) was also included. All tests were performed in triplicate.

### 2.3. Antifungal Activity

Five *M. pachydermatis* clinical isolates were tested in vitro for the antimicrobial sensitivity. The strains were previously cultured from skin of dogs with atopic dermatitis.

The antifungal activity of selected EOs was assessed by microdilution method, using liquid m-Dixon medium for preparing yeast suspensions, as reported elsewhere [23]. The yeasts were tested against ketoconazole by *E*-test (AB Biodisk, Solna, Sweden) to evaluate the efficacy of a conventional antimycotic drug, currently employed for the treatment of *Malassezia* infections [6].

The fungal isolates were tested in triplicate against the selected EOs diluted into the medium at concentrations of 5%, 4.5%, 4%, 3.5%, 3%, 2.5%, 2%, 1.5%, 1%, 0.75%, and 0.5%. MIC was established as the lowest concentration of EO where no fungal growth was yielded.

## 3. Results

### 3.1. Essential Oil Composition

Taken in consideration compounds present in percentage equal to or greater than 1% in at least one of the investigated EO, 49 compounds were identified (Table 1), representing 99.4%–100% of the total identified fraction. The oxygenated monoterpenes was the main class of compounds in seven out of nine species tested in this work (*A. triphylla*, *C. citratus*, *T. vulgaris*, *L. cubeba*, *S. montana*, *M. officinalis*, and *O. vulgare*), with a percentage ranging from 34.7% (*A. triphylla*) to 88.2% (*C. citratus*). *T.vulgaris* and *S. montana* shared the same amount of oxygenated monoterpenes and monoterpene hydrocarbons, with a slight predominance of oxygenated monoterpenes (53.1/35.2 and 47.6/39.8, respectively).

Among the Lamiaceae plants, only the *A. triphylla* sample differed from the others because monoterpene hydrocarbons were the main class of constituents (62.2%). Limonene (31.1%) and sabinene (26.0%) were the principal compounds.

Both of *L. cubeba* and *C. zeylanicum* belong to Lauraceae family, but they evidenced a great difference in their composition. In fact, *L. cubeba* EO was characterized by a high percentage of oxygenated monoterpene compounds (80.8%), while phenylpropanoids prevailed in *C. zeylanicum* EO (66.7%) where (*E*)-cinnamaldehyde (63.2%) was the main component.

The composition of *C. myrrha* EO showed good amount of oxygenated sesquiterpenes (63.1%) and sesquiterpenes hydrocarbons (36.0%) and pointed out furanoeudesma-1,3-diene as the major component (33.7%), followed by curzerene and lindestrene (17.5% and 11.9%, respectively). In more detail with respect to the EO composition, neral and geranial were the major compounds in *C. citratus*, *L. cubeba*, and *M. officinalis* with the respective percentages of 32.6%–40.1%, 31.8%–38.2% and 29.0%–36.5%.

*p*-Cymene showed the highest amount in *T. vulgaris* (19.4%) and *S. montana* (14.8%), although the most abundant constituent was thymol (40.5%) in *T. vulgaris* and carvacrol (38.2%) in *S. montana*. This latter compound was also the principal one in *O. vulgare* (66.5%).

### 3.2. Antibacterial Activity

#### 3.2.1. Agar Disk Diffusion Method

The nine EOs tested in this study showed different degrees of growth inhibition against the assayed *Staphylococcus* isolates. The strongest antibacterial activity was observed with *O. vulgare* and *T. vulgaris* EOs: the overall inhibition zone ranged from 9.0 to 13.0 mm and from 7.0 to 22.0 mm, respectively. On the other hand, the lowest activities were shown by *C. zeylanicum* and *C. myrrha* EOs.

*C. myrrha* EO was not active against the three *S. xylosus* isolates, and the remaining EOs showed weak effectiveness against them.

No growth inhibition zone was observed with DMSO as negative control, whereas chloramphenicol, included as positive control, resulted effective against all isolates. Results are summarized in Table 2.

#### 3.2.2. Minimum Inhibitory Concentration

The minimum inhibitory concentration (MIC) values, expressed both as percentage and mg/mL, testing EOs versus the *Staphylococcus* spp. isolates are reported in Table 3. *O. vulgare*, *T. vulgaris* and *S. montana* showed good antibacterial activity against all the selected strains, with MICs ranging from 0.29 to 0.58 mg/mL for *O. vulgare*, from 0.58 to 1.16 mg/mL for *T. vulgaris*, and from 0.56 to 1.12 mg/mL for *S. montana*.

*C. zeylanicum* and *C. myrrha* had the lowest antistaphylococcal activity with MIC of 10.2 mg/mL versus six isolates and of 10.0 mg/mL versus five isolates, respectively.

*A. triphylla* showed not high MICs varying from 2.17 to 8.7 mg/mL in relation to the tested isolate. The remaining EOs showed a weak activity: 0.55–2.23 mg/mL for *C. citratus*, 1.10–4.42 mg/mL for *L. cubeba*, and 1.11–2.22 mg/mL for *M. officinals*.

No growth inhibition was observed with the negative control, whereas chloramphenicol resulted active against all strains.

### 3.3. Antifungal Activity

Selected EOs showed different degrees of efficacy against *M. pachydermatis* isolates (Table 3). In detail, *C. myrrha* and *L. cubeba* were not effective at 5% dilution. *A. tryphilla* was the most active with MICs of 0.87 and 1.03 mg/mL, followed by *S. montana* with MIC of 1.8 mg/mL and *C. zeylanicum* with 3.06 and 4.08 mg/mL.

## 4. Discussion

The results obtained in the present survey showed different antimicrobial activity degrees in relation to the EO and the bacterial or fungal isolates assayed.

Different staphylococcal species, all previously isolated from dogs with skin infections, were examined in our study. Even though *S. pseudointermedius* is considered the primary canine cutaneous pathogen [24], other staphylococcal species may be involved in pyoderma and atopic dermatitis. In fact, bacteriological examinations for some canine clinical cutaneous cases allowed us to isolate, other than *S. pseudointermedius*, also *S. aureus*, *S. chromogenes*, *S. hyicus*, and *S. xylosus*, which are usually related to infections in other animal species.

*S. chromogenes,* a coagulase-negative species, causes mastitis in dairy animals [25]. *S. hyicus*, considered a coagulase-variable species, is mainly found in pigs, but it is also frequently isolated from canine specimens [26]. *S. xylosus* is a coagulase-negative staphylococcal species considered as commensal and able to colonize the skin of mammals and birds [27]. Moreover, it is reported as the most frequently isolated coagulase-negative staphylococcus from skin and mucous membrane of healthy dogs [28]. *S. aureus* is the main pathogen responsible for mastitis in dairy animals [29], as well as it is involved in infections of different anatomic districts in humans, as well as farm and companion animals [30]. Moreover, it is a major food poisoning microorganism posing risk to consumer health, mainly through its production of heat-stable enterotoxins [31].

*O. vulgare* and *T. vulgaris* EOs resulted very active against all staphylococcal strains tested in the present survey. These results are corroborated by other studies that found high antimicrobial activity against several bacterial species, including *Staphylococcus* spp. [11,12,32,33].

The antibacterial effect has been related to the major compounds of these two oils, in particular carvacrol and thymol [34,35]. Exposure of bacterial cells to carvacrol has resulted in increases in the membrane fluidity and leakage of protons and potassium ions, leading to a decrease in pH gradient across the cytoplasm membrane, a collapse of the membrane potential, an inhibition of ATP (adenosine triphosphate) synthesis, and ultimately cell death [36].

As regards thymol, it has been speculated that its antimicrobial effect may result, at least in part, from a perturbation of the lipid fraction of the bacterial plasma membrane resulting in the leakage of intracellular materials [37].

*S. montana* EO showed a very good anti-staphylococcal activity, too. Antimicrobial effectiveness of *S. montana* EO against some Gram-positive and Gram-negative bacteria was previously observed, and it was related to major compounds, such as carvacrol. In particular, Vitanza et al. [38] found that *S. aureus,* submitted to the action of *S. montana* EO, showed collapse of cell wall without breaks.

Our study confirmed thymol and carvacrol as main constituents of the EOs with the best antibacterial activity: 40.5% of thymol in *T. vulgaris* and 38.2% and 66.5% of carvacrol in *S. montana* and *O. vulgare*, respectively. Moreover, *T. vulgaris* and *S. montana* EOs had the highest amount of *p*-cymene (19.4% and 14.8%, respectively), a monoterpene compound with antimicrobial properties [39].

EOs from *C. myrrha* and *C. zeylanicum* showed the lowest activity against the bacterial isolates, mainly against the three *S. xylosus* strains. No relevant differences were observed testing *C. myrrha* and *C. zeylanicum* against the remaining staphylococcal isolates. In detail, *C. zeylanicum* was active against six isolates of the eight tested with high MIC value (10.2 mg/mL). This result is in contrast with the good antibacterial activity of *C. zeylanicum* EO previously observed against *S. aureus* [40]. Similarly, *C. myrrha* EO was effective against five of the tested isolates with 10 mg/mL MIC, and it was not active against the three tested *S. xylosus* strains. Mahboubi and Kazempour [41] found relevant activity of *C. myrrha* against a *S. aureus* ATCC strain, whereas Adam and Selim [42] observed slight sensitivity of *S. aureus* to myrrh oil.

*C. citratus*, *L. cubeba*, and *M. officinalis* EOs showed quite similar effectiveness. Anti-*S. aureus* activity of these EOs was previously reported by other authors. In particular, lemongrass and lemon balm were found more active against Gram-positive bacteria, such as *S. aureus*, than Gram-negative ones [43,44]. Litsea was demonstrated to be an effective bacterial inhibitor and bactericide against methicillin-resistant *S. *aureus** for a destructive effect on the bacterial cell membrane [45].

Scant information about the anti-staphylococcal activity of *A. tryphilla* is available; however, its effectiveness against reference *S. aureus* strains has been observed [46,47,48]. *A. triphylla* EO does not provoke whole cell lysis of *S. aureus* but compromises the structural integrity of the plasmic membrane and induces a loss of the cytoplasmic contents, with consequent cellular death [48].

*A. triphylla* EO showed a strong anti-*Malassezia* activity. To the best of our knowledge there are no studies regarding the activity of this EO against *Malassezia*. Nevertheless, lemon verbena EO has been reported as active against different fluconazole-resistant *Candida* spp. isolated from human patients, with MIC values higher (35–140 mg/mL) than the value observed in the present study. Moreover, this EO showed a good activity against *Aspergillus fumigatus* in a previous study [49] and a poor effectiveness versus the probiotic yeast *Saccharomyces cerevisiae* [50], indicating a variable efficacy against different species of both molds and yeasts. The antifungal activity seems to be related to a higher content of limonene and sabinene, in comparison with the other EOs. For these reasons *A. triphylla* EO would appear of great interest when used as antimycotic compound, paying attention to the fungal species involved.

*S. montana* showed MIC values of 1.8 mg/mL, resulting more effective in comparison with the well-known antimycotic compounds from *O. vulgare* and *T. vulgaris*. These results are not in agreement with a previous study [51], where *O. vulgare* showed a very low MIC against malassezia isolates from canine dermatitis.

This finding is of interest, considering that this EO also appeared active against staphylococci. *S. montana* has been recently reported as active against *M. pachydermatis* recovered from canine otitis [52], *Candida albicans* [53], and *Candida glabrata* [54] and moderately effective against dermatophytes [55], suggesting a good activity against yeasts.

Another interesting feature is the high sensitivity of *Malassezia* to *C. zeylanicum.* These results are in agreement with findings reported by Bismarck et al. [52] and Sim et al. [56] in otologic canine isolates. This EO, in fact, although poorly effective against molds such as *A. fumigatus* [49] and dermatophytes [55], showed a strong antimicrobial activity versus *Salmonella enterica* serotype Typhimurium and *Escherichia coli* isolated from poultry [50].

On the other hand, *Malassezia* yeasts showed a marked variability in their sensitivity to EOs. For these reasons, a sensitivity assay of the fungal isolates is recommended, as suggested by Bismarck et al. [52].

## 5. Conclusions

The overuse of antibiotics has led to the extensive antibiotic resistance in pathogenic bacteria, including staphylococci, of human and veterinary concern. In this view, natural products such as EOs with antimicrobial properties could represent a suitable alternative in the treatment of infections, mainly when conventional drugs resulted not effective.

Our results underlined and corroborated the variability of the EOs’ activity in relation not only to the bacterial species, but also to the isolates [10]. Consequently, there is not always a correspondence between results obtained with reference and wild strains. Even though the antimicrobial activity of a given EO has been previously determined, an in vitro antibacterial/antifungal sensitivity test should always be performed to better verify the effectiveness of the EO against the studied strains.

Our in vitro study showed the activity of *O. vulgare* and *T. vulgaris* EOs against cutaneous staphylococcal isolates, and the good effectiveness of *S. montana* EO against both staphylococcal and *M. pachydermatis* strains. After a proper in vivo evaluation, these EOs could be a promising treatment to combat canine cutaneous mixed infections due to these pathogens.

To the best of our knowledge, this is the first study that found, among EOs of which antimicrobial activity has already been defined, the natural product active versus both staphylococcal and *Malassezia* strains involved in canine cutaneous infections.

## Figures and Tables

**Table 1:** Relative percentage of the main constituents of the tested essential oils.

Compounds	L.R.I.	Class	EO-1	EO-2	EO-3	EO-4	EO-5	EO-6	EO-7	EO-8	EO-9
α-Thujene	930	mh	0.2		1.5		2.0				1.6
α-Pinene	939	mh	1.1	0.2	0.9	1.2	1.4		0.3		0.7
Camphene	954	mh		0.8	0.8	0.2	0.5		0.1		0.2
Benzaldheyde	960	nt							0.2		
Sabinene	975	mh	26.0			0.8					
β-Pinene	979	mh				0.8			0.1		
Oct-1-en-3-ol	979	nt	0.1		0.7		1.9	0.3			0.6
Octan-3-one	984	nt									0.2
Methyl heptenone	986	nt	0.2	1.5		1.3		1.0			
Myrcene	991	mh	0.5		1.6	0.3	1.8	0.2			1.8
Dehydro-1,8-cineole	991	om		0.1							
*n*-Octanal	999	nt		0.1							
α-Phellandrene	1003	mh			0.2		0.4		0.6		0.3
α-Terpinene	1017	mh			1.3		2.6		0.3		1.4
*p*-Cymene	1025	mh	0.5		19.4	0.2	14.8		1.3		6.0
Limonene	1029	mh	31.1	1.5	0.6	12.2	1.6	0.1		0.4	0.5
β-Phellandrene	1030	mh							1.7		
δ-3-Carene	1031	mh			0.1		0.1				0.1
1,8-Cineole	1031	om	6.1	0.2	0.4	1.4	0.7				
(*Z*)-β-Ocimene	1037	mh	0.1	0.1			0.1				
(*E*)-β-Ocimene	1050	mh	2.4				0.1	0.2			
Bergamal	1057	nt	0.3								
γ-Terpinene	1060	mh	0.2		8.6		14.2				6.8
*cis*-Sabinene hydrate	1070	om	0.3		0.5		0.6				0.5
Allyl hexanoate	1076	nt		0.9							
Terpinolene	1089	mh			0.2		0.2				0.1
Rosefuran	1093	om	0.1								
α-Pinene oxide	1095	om						0.2			
Linalool	1097	om	3.4	1.1	2.4	1.5	1.4	0.3	3.1		0.2
*cis*-*p*-Mentha-2,8-dien-1-ol	1102	om		0.2							
α-Thujone	1102	om	0.4								
*cis*-Rose oxide	1108	om						0.1			
β-Thujone	1114	om	0.3								
*trans*-*p*-Mentha-2,8-dien-1-ol	1123	om	0.5								
*cis*-Limonene oxide	1137	om	0.5	0.2							
*trans*-Limonene oxide	1142	om	0.2								
*cis*-Sabinol	1143	om	0.8								
Camphor	1146	om			0.7	0.1	0.1				
Isopulegol	1150	om	0.2					0.2			
Citronellal	1153	om	11.2	0.6		1.5		8.1			
*trans*-Chrysanthemal	1153	om		0.3				0.8			
Hydrocinnamaldehyde	1162	nt							0.3		
*cis*-Chrysanthenol	1164	om				0.5					
Borneol	1169	om			1.4		1.7				0.3
Lavandulol	1170	om						0.1			
Isoneral	1170	om		0.8				1.2			
Rosefuran epoxide	1176	om		0.2							
4-Terpineol	1177	om	0.8		1.4	0.2	1.2		0.2		0.7
*p*-Cymen-8-ol	1183	om					0.1				
Isogeranial	1185	om		1.1		0.8		1.8			
α-Terpineol	1189	om	0.6	0.2	0.2	0.6	0.2		0.6		0.1
Dihydro carveol	1194	om	0.1	0.2				0.1			
*n-*Decanal	1202	nt		0.3							
*trans*-Isopiperitenol	1210	om	1.5								
*trans*-Carveol	1217	om	0.5								
(*Z*)-Cinnamaldehyde	1219	nt							0.8		
Citronellol	1226	om	3.3	0.5				2.0			
Nerol	1230	om				0.5					
Thymyl methyl ether	1235	om			0.5						
Neral	1238	om	0.8	32.6		31.8		29.0			
Carvone	1243	om	0.5								
Carvacrol methyl ether	1244	om			1.6		1.2				
Geraniol	1253	om	0.1	5.0		1.0		1.8			
Methyl citronellate	1261	om						0.4			
Geranial	1267	om	1.4	40.1		38.2		36.5			
(*E*)-Cinnamaldehyde	1270	nt							63.2		
*cis*-Pulegone oxide	1275	om		0.3		0.2		0.1			
(*Z*)-Methyl geranate	1279	nt						0.2			
3,4-Diethylphenol	1284	om			0.2						
Thymol	1290	om			40.5		1.6				6.8
Carvacrol	1299	om			0.2	2.2	38.2				66.5
(*E*)-Cinnamyl alcohol	1304	nt							0.1		
2-Ethyl-4,5-dimethyl-Phenol	1305	om			3.1						
6,10-Dimethyl-Dodeca-1,6-dien-12-ol	1318	om		0.1		0.3					
Methyl geranate	1325	om						0.7			
δ-Elemene	1338	sh								0.7	
Citronellyl acetate	1354	om	0.4	0.1							
Thymyl acetate	1355	om			0.1						
Eugenol	1359	pp							3.5		
Neryl acetate	1362	om						0.2			
Carvacrol acetate	1373	om					0.5				
α-Copaene	1377	sh	0.2			0.5	0.2	0.1	0.7		
Geranyl acetate	1381	om	0.6	4.5				1.7			
β-Bourbonene	1388	sh					0.1			0.5	
β-Elemene	1391	sh								6.9	
β-Caryophyllene	1419	sh		2.4							
β-Caryophyllene	1419	sh	1.5		5.8	0.9	4.6	9.0	6.2	0.4	3.6
γ-Elemene	1437	sh								0.4	
Cinnamyl acetate	1445	nt							3.5		
(*E*)-Isoeugenol	1451	pp		0.2							
α-Humulene	1455	sh		0.2	0.2		0.2	0.5	1.2	0.2	0.2
Alloaromadendrene	1460	sh	0.2								
γ-Muurolene	1480	sh			0.4		0.3				
Germacrene D	1485	sh					0.6	1.5		1.5	
β-Selinene	1490	sh								0.9	
2-Isopropyl-4α,8-dimethyl-1,2,3,4,4α,5,6,7-octahydronaphthalene	1491	sh								0.2	
α-Selinene	1494	sh								0.8	
Valencene	1496	sh			0.1		0.2				
Curzerene	1499	sh								17.5	
Bicyclogermacrene	1500	sh						0.1			
α-Bulnesene	1505	sh								0.8	
β-Bisabolene	1506	sh					2.7				0.5
γ-Cadinene	1513	sh							6.2		
*trans*-γ-Cadinene	1514	sh		1.6	0.6		0.2				
δ-Cadinene	1523	sh		0.3	0.6		0.5	0.1	0.2	0.2	
(*E*)-Ortho-methoxy cinnamaldehyde	1529	nt							0.5		
(E)-γ-Bisabolene	1531	sh		0.1							
Nerolidol	1534	os		0.2							
Elemol	1550	os								0.5	
*p*-Cymene-2,5-diol	1555	om					0.2				
Germacrene B	1561	sh								5.2	
Germacrene D-4-ol	1576	os						0.1			
Spathulenol	1578	os					0.2				
Caryophyllene oxide	1583	os		1.0	2.7	0.2	1.1	0.6	1.3		0.3
Furanoeudesma-1,4-diene	1587	os								0.7	
Humulene epoxide II	1608	os							0.1		
Tetradecanal	1613	nt							0.2		
epi-α-Cadinol	1640	os			0.5						
Furanoeudesma-1,3-diene	1645	os								33.7	
Lindestrene	1652	os								11.9	
Atractylone	1669	os								9.8	
(*Z*)-α-santalol	1681	os			0.1						
Germacrone	1694	os								1.0	
Cyclodeca[b]furan, 4,7,8,11-tetrahydro-8-methoxy-3,6,10-trimethyl-, (5E,8R,9E)-	1733	os								5.6	
Benzyl benzoate	1760	nt							2.6		
Isovaleric acid, dodecyl ester	1845	nt							0.3		
6-methyl-4,6-bis(4-methylpent-3-en-1-yl)cyclohexa-1,3-dienecarbaldehyde	2113	od		0.1							
*trans*-Geranylgeraniol	2201	od				0.2					
Isobutyl angelate	1045	nt						0.3			
2-Methyl-2-decanol	1231	nt	0.2								
2,6-Dimethyl-5-hepten-1-ol		nt	0.5								
Unknown			0.0	0.3	0.0	0.4	0.1	0.4	0.6	0.5	0.0
**Class of Compounds**			**EO-1**	**EO-2**	**EO-3**	**EO-4**	**EO-5**	**EO-6**	**EO-7**	**EO-8**	**EO-9**
Monoterpene Hydrocarbons (mh)			62.2	2.6	35.2	15.7	39.8	0.5	4.3	0.4	19.5
Oxygenated monoterpenes (om)			34.7	88.2	53.1	80.8	47.6	85.3	3.9	0.0	75.0
Sesquiterpene Hydrocarbons (sh)			1.8	4.6	7.6	1.5	9.5	11.3	14.5	36.0	4.3
Oxygenated Sesquiterpenes (os)			0.0	1.2	3.3	0.2	1.2	0.7	1.4	63.1	0.3
Oxygenated diterpenes			0.0	0.1	0.0	0.2	0.0	0.0	0.0	0.0	0.0
Phenylpropanoids (pp)			0.0	0.2	0.0	0.0	0.0	0.0	66.7	0.0	0.0
Non-terpene derivatives (nt)			1.3	2.8	0.7	1.3	1.9	1.8	8.5	0.0	0.9
**Total Identified**			**100.0**	**99.7**	**100.0**	**99.6**	**99.9**	**99.6**	**99.3**	**99.5**	**100.0**

L.R.I.: Linear Retention Index; EO-1: *Aloysia triphylla*; EO-2: *Cymbopogon citratus*; EO-3: *Thymus vulgaris*; EO-4: *Litsea cubeba*; EO-5: *Satureja montana*; EO-6: *Melissa officinalis*; EO-7: *Cinnamomum zeylanicum*; EO-8: *Commiphora myrrha*; EO-9: *Origanum vulgare*; mh: monoterpene hydrocarbons; om: oxygenated monoterpenes; sh: sesquiterpene hydrocarbons; os: oxygenated sesquiterpenes; nt: non-terpenes; pp: phenylpropanoids; od: oxygenated diterpenes.

**Table 2:** The growth inhibition zones (expressed in mm) obtained testing the selected *Staphylococcus* isolates against the assayed EOs.

Bacterial Strain	Essential Oil									CF
	*Aloysia triphylla*	*Cinnamomum zeylanicum*	*Commiphora myrrha*	*Cymbopogon citratus*	*Litsea cubeba*	*Melissa officinalis*	*Origanum vulgare*	*Satureja montana*	*Thymus vulgaris*	
	M ± SD	M ± SD	M ± SD	M ± SD	M ± SD	M ± SD	M ± SD	M ± SD	M ± SD	
*Staphylococcus chromogenes* 3	8.0 ± 0.0	7 ± 0.0	7 ± 0.0	8.0 ± 1.0	8.0 ± 0.0	8.0 ± 0.0	13 ± 0.0	8.0 ± 0.0	9 ± 0.6	21 (S)
*Staphylococcus chromogenes* 42	8.0 ± 0.6	6 ± 0.0	6 ± 0.0	8.0 ± 0.0	8.0 ± 1.0	8.0 ± 0.6	10 ± 0.6	9 ± 0.0	9 ± 0.0	18 (S)
*Staphylococcus aureus* 22	8.0 ± 0.0	7 ± 1.0	7 ± 0.6	7.0 ± 1.0	8.0 ± 0.0	7.0 ± 0.0	11 ± 1.0	8 ± 1.0	22 ± 0.6	21 (S)
*Staphylococcus pseudointermedius* 15	7.0 ± 0.0	7.0 ± 0.0	7 ± 0.0	8.0 ± 0.0	8.0 ± 0.6	7.0 ± 1.0	10 ± 0.0	8 ± 1.0	8 ± 0.0	19 (S)
*Staphylococcus hyicus* 129	7.0 ± 0.6	7.0 ± 0.6	8 ± 0.0	8.0 ± 0.6	8.0 ± 1.0	8.0 ± 0.0	13 ± 0.0	11 ± 0.6	10 ± 1.0	20 (S)
*Staphylococcus xylosus* 191	7.0 ± 0.0	7.0 ± 0.0	0 ± 0.0	7.0 ± 0.0	8.0 ± 0.0	7.0 ± 0.6	12 ± 0.0	8 ± 0.6	8 ± 0.0	18 (S)
*Staphylococcus xylosus* 214	6.0 ± 0.0	0.0 ± 0.0	0 ± 0.0	0.0 ± 0.0	7.0 ± 1.0	6.0 ± 0.0	9 ± 0.0	7.0 ± 0.0	7 ± 0.0	19 (S)
*Staphylococcus xylosus* 231	7.0 ± 0.0	0.0± 0.0	0 ± 0.0	8.0 ± 0.0	8.0 ± 0.0	6.0 ± 0.0	10 ± 0.6	8.0 ± 0.0	8 ± 0.6	20 (S)

M: mean expressed in mm; SD: standard deviation; CF: chloramphenicol; S: susceptible.

**Table 3:** MIC values of the tested EOs expressed in percentage and mg/mL against selected *Staphylococcus* spp. and *Malassezia pachydermatis* isolates.

Bacterial Strain	Essential Oil																		CF
	*Aloysia triphylla*		*Cinnamomum zeylanicum*		*Commiphora myrrha*		*Cymbopogon citratus*		*Litsea cubeba*		*Melissa officinalis*		*Origanum vulgare*		*Satureja montana*		*Thymus vulgaris*		
	%	mg/mL	%	mg/mL	%	mg/mL	%	mg/mL	%	mg/mL	%	mg/mL	%	mg/mL	%	mg/mL	%	mg/mL	µg/mL
*Staphylococcus chromogenes* 3	*1.25*	*2.17*	5	10.2	5	10	0.6	1.11	2.5	4.42	1.25	2.22	0.15	0.29	0.6	1.12	0.6	1.16	8
*Staphylococcus chromogenes* 42	1.25	2.17	5	10.2	5	10	0.3	0.55	1.25	2.21	1.25	2.22	0.15	0.29	0.3	0.56	0.3	0.58	6
*Staphylococcus aureus* 22	1.25	2.17	5	10.2	5	10	0.6	1.11	0.6	1.10	1.25	2.22	0.15	0.29	0.3	0.56	0.3	0.58	6
*Staphylococcus pseudointermedius* 15	1.25	2.17	5	10.2	5	10	1.25	2.23	1.25	2.21	1.25	2.22	0.15	0.29	0.3	0.56	0.3	0.58	8
*Staphylococcus hyicus* 129	2.5	4.35	5	10.2	5	10	1.25	2.23	1.25	2.21	0.6	1.11	0.15	0.29	0.3	0.56	0.3	0.58	7
*Staphylococcus xylosus* 191	2.5	4.35	5	10.2	ne	ne	1.25	2.23	1.25	2.21	1.25	2.22	0.3	0.58	0.3	0.56	0.3	0.58	7
*Staphylococcus xylosus* 214	2.5	4.35	ne	ne	ne	ne	1.25	2.23	1.25	2.21	1.25	2.22	0.3	0.58	0.3	0.56	0.3	0.58	8
*Staphylococcus xylosus* 231	5	8.7	ne	ne	ne	ne	1.25	2.23	1.25	2.21	0.6	1.11	0.3	0.58	0.3	0.56	0.3	0.58	6
*Malassezia pachydermatis* 1	0.75	1.03	1.5	3.06	ne	ne	4	7.13	ne	ne	2	3.55	4	7.73	1	1.8	4.5	8.7	* 0.02
*Malassezia pachydermatis* 2	0.5	0.87	1.5	3.06	ne	ne	4	7.13	ne	ne	2	3.55	4	7.73	1	1.8	4.5	8.7	* 0.02
*Malassezia pachydermatis* 3	0.75	1.03	2	4.08	ne	ne	4	7.13	ne	ne	2	3.55	3.5	6.76	1	1.8	4	7.73	* 0.02
*Malassezia pachydermatis* 4	0.5	0.87	1.5	3.06	ne	ne	4	7.13	ne	ne	2	3.55	4	7.73	1	1.8	4	7.73	* 0.02
*Malassezia pachydermatis* 5	0.75	1.03	2	4.08	ne	ne	4	7.13	ne	ne	1.5	2.66	3.5	6.76	1	1.8	4	7.73	* 0.02

ne: not effective; CF: chloramphenicol; * ketoconazole.

## References

[1] Santoro D. (2019). Therapies in canine atopic dermatitis: An update. Vet. Clin. North Am. Small Anim. Pract..

[2] Chermprapai S., Ederveen T.H.A., Broere F., Broens E.M., Schlotter Y.M., Schalkwijk S., Boekhorst J., van Hijum A.F.T., Rutten V.P.M.G. (2019). The bacterial and fungal microbiome of the skin of healthy dogs and dogs with atopic dermatitis and the impact of topical antimicrobial therapy, an exploratory study. Vet. Microbiol..

[3] Reddy B.S., Kumari K.N., Rao V.V., Rayulu V.C. (2014). Efficacy of cefpodoxime with clavulanic acid in the treatment of recurrent pyoderma in dogs. ISRN Vet. Sci..

[4] Bjerre R.D., Bandier J., Skov L., Engstrand L., Johanses J.D. (2017). The role of the skin microbiome in atopic dermatitis: A systematic review. Br. J. Dermatol..

[5] De Boer D.J., Marsella R. (2001). The ACVD task force on canine atopic dermatitis (XII): The relationship of cutaneous infections to the pathogenesis and clinical course of canine atopic dermatitis. Vet. Immunol. Immunopathol..

[6] Nijima M., Kano R., Nagata M., Hasegawa A., Kamata H. (2011). An azole-resistant isolate of *Malassezia pachydermatis*. Vet. Microbiol..

[7] Watanabe S., Koike A., Kano R., Nagata M., Chen C., Hwang C.Y., Hasegawa A., Kamata H. (2014). In vitro susceptibility of *Malassezia pachydermatis* isolates from canine skin with atopic dermatitis to ketoconazole and itraconazole in East Asia. J. Vet. Med. Sci..

[8] Dhifi W., Bellili S., Jazi S., Bahloul N., Mnif W. (2016). Essential oils’ chemical characterization and investigation of some biological activities: A critical review. Medicines.

[9] Artini M., Patsilinakos A., Papa R., Bozovic M., Sabatino M., Garzoli S., Vrenna G., Tilotta M., Pepi F., Ragno R. (2018). Antimicrobial and antibiofilm activity and machine learning classification analysis of essential oils from different mediterranean plants against pseudomonas aeruginosa. Molecules.

[10] Patsilinakos A., Artini M., Papa R., Sabatino M., Božovic M., Garzoli S., Vrenna G., Buzzi R., Manfredini S., Selan L. (2019). Machine learning analyses on data including essential oil chemical composition and in vitro experimental antibiofilm activities against *Staphylococcus* species. Molecules.

[11] Kot B., Wierzchowska K., Piechota M., Czerniewicz P., Chrznowski G.C. (2019). Antimicrobial activity of five essential oils from lamiaceae against multidrug-resistant *Staphylococcus aureus*. Nat. Prod. Res..

[12] Sakkas H., Economou V., Gousia P., Bozidis P., Sakkas V., Petsios S., Mpekoulis G., Ilia A., Papadopoulou C. (2018). Antibacterial efficacy of commercially available essential oils tested against drug.resisitant Gram-positive pathogens. Appl. Sci..

[13] Adams R.P. (1995). Identification of Essential Oil Components by Gas Chromatography/Quadrupole Mass Spectroscopy.

[14] Davies N.W. (1990). Gas chromatographic retention indices of monoterpenes and sesquiterpenes on Methyl Silicon and Carbowax 20M phases. J. Chromatogr. A.

[15] Jennings W., Shibamoto T. (1982). Qualitative Analysis of Flavor and Fragrance Volatiles by Glass Capillary Gas Chromatography, Food/Nahrung.

[16] Masada Y. (1976). Analysis of Essential Oils by Gas Chromatography and Mass Spectrometry.

[17] Stenhagen E., Abrahamsson S., McLafferty F.W. (1974). Registry of Mass Spectral Data.

[18] Swigar A.A., Silverstein R.M. (1981). Monoterpenes.

[19] CLSI (2012). Performance Standards for Antimicrobial Disk Susceptibility Tests; Approved Standard.

[20] National Committee for Clinical Laboratory Standards (2002). Performance Standards for Antimicrobial Susceptibility Testing. Twelfth International Supplement.

[21] CLSI—National Committee for Clinical Laboratory Standards (1990). Methods for Dilution Antimicrobial Susceptibility Tests for Bacteria That Grow Aerobically.

[22] Ebani V.V., Nardoni S., Bertelloni F., Giovanelli S., Rocchigiani G., Pistelli L., Mancianti F. (2016). Antibacterial and antifungal activity of essential oils against some pathogenic bacteria and yeasts shed from poultry. Flav. Fragr. J..

[23] Nardoni S., Pistelli L., Baronti I., Najar B., Pisseri F., Bandeira Reidel R.V., Papini R., Perrucci S., Mancianti F. (2017). Traditional Mediterranean plants: Characterization and use of an essential oils mixture to treat *Malassezia* otitis externa in atopic dogs. Nat. Prod. Res..

[24] Bannoehr J., Guardabassi L. (2012). *Staphylococcus pseudintermedius* in the dog: Taxonomy, diagnostics, ecology, epidemiology and pathogenicity. Vet. Dermatol..

[25] Vanderhaeghen W., Piepers S., Leroy F., Van Coillie E., Haesebrouck F., De Vliegher S. (2014). Effect, persistence, and virulence of coagulase-negative *Staphylococcus* species associated with ruminant udder health. J. Dairy Sci..

[26] Conner J.G., Smith J., Erol E., Locke S., Phillips E., Carter C.N., Odoi A. (2018). Temporal trends and predictors of antimicrobial resistance among *Staphylococcus* spp. isolated from canine specimens submitted to a diagnostic laboratory. PLoS ONE.

[27] Dordet-Frisoni E., Dorchies G., De Araujo C., Talon R., Leroy S. (2007). Genomic diversity in Staphylococcus xylosus. Appl. Environ. Microbiol..

[28] Cox H.U., Hoskins J.D., Newman S.S., Foil C.S., Turnwald G.H., Roy A.F. (1988). Temporal study of staphylococcal species on healthy dogs. Am. J. Vet. Res..

[29] Barkema H.W., Schukken Y.H., Zadoks R.N. (2006). Invited Review: The role of cow, pathogen, and treatment regimen in the therapeutic success of bovine *Staphylococcus aureus* mastitis. J. Dairy Sci..

[30] Lowy F.D. (1998). Staphylococcus aureus infections. N. Engl. J. Med..

[31] Bore E., Langsrud S., Langsrud O., Mari Rode1 T., Holck A. (2007). Acid-shock responses in *Staphylococcus aureus* investigated by global gene expression analysis. Microbiology.

[32] de Oliveira J.L.T., Diniz M.F.M., de Oliveira Lima E., Souza E.L., Trajano V.N., Santos B.H.C. (2009). Effectiveness of *Origanum vulgare* L. and *Origanum majorana* L. essential oils in inhibiting the growth of bacterial strains isolated from the patients with conjunctivitis. Braz. Arch. Biol. Technol..

[33] Ebani V.V., Nardoni S., Bertelloni F., Najar B., Pistelli L., Mancianti F. (2017). Antibacterial and antifungal activity of essential oils against pathogens responsible for otitis externa in dogs and cats. Medicines.

[34] Burt S. (2004). Essential oils: Their antibacterial properties and potential applications in foods—A review. Int. J. Food Microbiol..

[35] Barros J.C., De Conceição M.L., Da Gomes Neto N.J., Costa C.V., Da Siqueira Júnior J.P., Basílio Junior I.D., De Souza E.L. (2009). Interference of *Origanum vulgare* L. essential oil on the growth and some physiological characteristics of *Staphylococcus aureus* strains isolated from foods. LWT—Food Sci. Technol..

[36] Ultee A., Gorris L.G.M., Smid E.J. (1998). Bactericidal activity of carvacrol towards the food-borne pathogen *Bacillus cereus*. J. Appl. Microbiol..

[37] Trombetta D., Castelli F., Sarpietro M.G., Venuti V., Cristani M.T., Daniele C., Saija A., Mazzanti G., Bisignano G. (2005). Mechanisms of antibacterial action of three monoterpenes. Antimicrob. Agents Chemother..

[38] Vitanza L., Maccelli A., Marazzato M., Scazzocchio F., Comanducci A., Fornarini S., Crestoni M.E., Filippi A., Fraschetti C., Rinaldi F. (2019). *Satureja montana* L. essential oil and its antimicrobial activity alone or in combination with gentamicin. Microb. Pathog..

[39] Marchese A., Arciola C.R., Barbieri R., Silva A.S., Nabavi S.F., Tsetegho Sokeng A.J., Izadi M., Jafari N.J., Suntar I., Daglia M. (2017). Update on monoterpenes as antimicrobial agents. A particular focus on p-cymene. Materials.

[40] Trajano V.N., Lima E.O., Travassos A.E., Souza E.L. (2010). Inhibitory effect of the essential oil from *Cinnamomum zeylanicum* blume leaves on some food-related bacteria. Ciênc. Tecnol. Aliment..

[41] Mahboubi M., Kazempour N. (2016). The antimicrobial and antioxidant activities of *Commiphora molmol* extracts. Biharean. Biol..

[42] Adam M.E., Selim S.A. (2013). Antimicrobial activity of essential oil and methanol extract from *Commiphora molmol* (Engl.) resin. Int. J. Curr. Microbiol. Appl. Sci..

[43] Naik M.I., Fomda B.A., Jaykumar E., Bhat J.A. (2010). Antibacterial activity of lemongrass (*Cymbopogon citratus*) oil against some selected pathogenic bacteria. Asian Pac. J. Trop. Med..

[44] Ehsani A., Alizadeh O., Hashemi M., Afshari A., Aminzare M. (2017). Phytochemical, antioxidant and antibacterial properties of *Melissa officinalis* and *Dracocephalum moldavica* essential oils. Vet. Res. Forum..

[45] Hu W., Li C., Dai J., Cui H., Lin L. (2019). Antibacterial activity and mechanism of *Litsea cubeba* essential oil against methicillin-resistant *Staphylococcus aureus* (*MRSA*). Ind. Crop. Prod..

[46] Demo M., Oliva M., Lopez M.L., Zunino M.P., Zygadlo J.A. (2005). Antimicrobial Activity of Essential Oils Obtained from Aromatic Plants of Argentina. J. Pharm. Biol..

[47] Sartoratto A., Machado A.L.M., Delarmelina C., Figueira G.M., Duarte M.C.T., Rehder V.L.G. (2004). Composition and antimicrobial activity of essential oils from aromatic plants used in Brazil. Braz. J. Microbiol..

[48] Oliva M.d.l.M., Carezzano E., Gallucci N., Freytes S., Zygadlo J., Demo M.S. (2015). Growth inhibition and morphological alterations of Staphylococcus aureus caused by the essential oil of *Aloysia triphylla*. B. Latinoam Caribe Pl..

[49] Ebani V.V., Najar B., Bertelloni F., Pistelli L., Mancianti F., Nardoni S. (2018). Chemical composition and in vitro antimicrobial efficacy of sixteen essential oils against *Escherichia coli* and *Aspergillus fumigatus* isolated from poultry. Vet. Sci..

[50] Ebani V.V., Nardoni S., Bertelloni F., Tosi G., Massi P., Pistelli L., Mancianti F. (2019). In vitro antimicrobial activity of essential oils against Salmonella enterica serotypes Enteritidis and Typhimurium strains isolated from poultry. Molecules.

[51] Nardoni S., Mugnaini L., Pistelli L., Leonardi M., Sanna V., Perrucci S., Pisseri F., Mancianti F. (2014). Clinical and mycological evaluation of an herbal antifungal formulation in canine Malassezia dermatitis. J. Mycol. Med..

[52] Bismarck D., Dusold A., Heusinger A., Muller E. (2019). Antifungal in vitro activity of essential oils against clinical isolates of *Malassezia pachydermatis* from canine ears: A report from a practice laboratory. Complement. Med. Res..

[53] Bona E., Cantamessa S., Pavan M., Novello G., Massa N., Rocchetti A., Berta G., Gamalero E. (2016). Sensitivity of *Candida albicans* to essential oils: Are they an alternative to antifungal agents?. J. Appl. Microbiol..

[54] Massa N., Cantamessa S., Novello G., Ranzato E., Martinotti S., Pavan M., Rocchetti A., Berta G., Gamalero E., Bona E. (2018). Antifungal activity of essential oils against azole-resistant and azole-susceptible vaginal *Candida glabrata* strains. Can. J. Microbiol..

[55] Nardoni S., Giovanelli S., Pistelli L., Mugnaini L., Profili G., Pisseri F., Mancianti F. (2015). In vitro activity of twenty commercially available, plant-derived essential oils against selected dermatophyte species. Nat. Prod. Commun..

[56] Sim J.X., Khazandi M., Pi H., Venter H., Trott D.J., Deo P. (2019). Antimicrobial effects of cinnamon essential oil and cinnamaldehyde combined with EDTA against canine otitis externa pathogens. J. Appl. Microbiol..

